# Case of Dr. John Stoughton Wolcott, of Litchfield, Conn., Son of the Late Oliver Wolcott, for Many Years Governor of That State, and Grandson of Oliver Wolcott, One of the Signers of the Declaration of Independence

**Published:** 1844-03

**Authors:** Solyman Brown


THE AMERICAN

### JOURNAL OF DENTAL SCIENCE.

Vol. IV.] MARCH, 1844. [No. 3.

### ARTICLE I.

*Case of Dr. John Stoughton Wolcott, of Litchfield, Conn., son of the late Oliver Wolcott, for many years Governor of that State, and grandson of Oliver Wolcott, one of the Signers of the Declaration of Independence.* By Solyman Brown, M. D., D. D. S.

The recent announcement, in the public newspapers and medical magazines, as well as in this Journal, of the death of this gentleman, in the outset of his professional career, *"by the application of arsenic to the nerve of a tooth,"* was well calculated to attract the attention of the writer, who was once the private tutor of Dr. Wolcott, and his fellow townsman. As one of the editors of the only Journal now in existence devoted exclusively to the interests of Dental Science, I felt it to be my duty to inquire into the facts of the case, for the purpose of making them known to the profession, for the common benefit of the human family. I felt convinced that the real facts connected with the melancholy demise of my early friend, were particularly important to my professional brethren, and to the public who employ their skill for the relief of one of the most intolerable of mortal maladies—THE TOOTH-ACHE. Under these impulses I addressed letters of inquiry to two physicians of acknowledged skill and reputation, giving a list of interrogatories, to which I solicited categorical replies. Both these gentlemen, Dr. R. M. Woodruff and Dr. J. G. Beckwith, in a manner which evinces their regard, not only for the truth of science, but for the welfare of humanity, made prompt and satisfactory replies.

If one of these gentlemen has sedulously avoided an expression of his private opinion as to the true cause of Dr. Wolcott's death, it may be ascribed either to a desire to give *facts* and *symptoms*, in order that gentlemen of the dental and medical professions might draw their own conclusions; or to a modesty which is equally creditable both to his head and heart. He might, moreover, have been unprepared, at the time, to contradict the opinion of his patient, on the one hand, or to inculpate the dental operator who had applied the arsenic to the tooth, on the other. I shall lay before the profession the communications furnished by these gentlemen, and by other physicians of the village of Litchfield, in the order in which they were received, taking this opportunity of tendering to them my sincere thanks, as Editor of the American Journal of Dental Science, and Secretary of the American Society of Dental Surgeons, for their prompt and laborious kindness in furnishing the following facts and documents in the case.

*Litchfield, Dec.* 13*th*, 1843.

Dr. Brown,

Sir—Your letter to my brother, Geo. C. Woodruff, Esq., requesting "the facts connected with the sudden decease of Dr. John S. Wolcott," has been handed me to answer. In reply, I will first state that for some time previous to the doctor's last illness, his health had been quite good; indeed, he remarked to me some eight or ten days before his attack, "that his health never was better." And upon inquiry I am informed by his nephew, (who resided with him,) and also by his housekeeper, that "until he first complained of tooth-ache he enjoyed perfect health;" so far as they could judge: he ate and slept well, made no complaint, and exhibited no symptom of the least ill health. The Friday evening previous to his attack, I was for some time with him; he was in fine spirits and apparently in good health. On Saturday following, he also appeared the same, and it has been remarked since his death, by some who spent an hour or more with him Saturday evening, that "they seldom saw him more cheerful than he was at that time." I have been somewhat particular in my inquiries respecting the doctor's health previous to his attack of tooth-ache, for the purpose of ascertaining or satisfying myself whether the symptoms which were presented during his illness, could be attributed to any cause existing previously: and from all the information which I have obtained, I have no doubt, that until Saturday evening, November 18th, he was in *good health,* and showed no symptoms which could be supposed to have been the cause of his illness. On this evening, (November 18th,) he retired at his usual hour, about half past nine o'clock, and after remaining in bed about one hour, he requested his nephew, who slept in an adjoining room, "to get him some laudanum, as his tooth ached," he applied the laudanum to his tooth, and soon after fell asleep, and slept till morning. On Sunday morning he arose early, ate his breakfast as usual, made no complaint, and went to his office. At about nine o'clock, he again complained of his tooth, but soon went out to visit his patients: about noon he returned to his house, said, "his tooth ached, and he had been to the dentists to have it taken out, but he did not pull it, but put something into it." He sat down to dinner, and after eating a few mouthfuls, he asked for a *pin*. He applied the pin to his tooth, and when he took it from his mouth there was something white on the point; he laid the pin and what he took from his tooth on the table, which one of his family examined after the doctor left, and said "it looked like wax." He ate but little dinner, left the table, and went to bed, and remained there until about three o'clock, P. M., when he got up and again complained of his tooth, and continued doing so until about seven in the evening, when he again went to the dentist and had the tooth extracted, which gave immediate relief. He returned to his house and went to bed at his usual hour, and lay there through the night. On Monday he arose earlier than usual, his face a little swollen, appeared to feel quite anxious about something, looked in the mirror often, ate but little breakfast, made no complaint, went out visiting patients, told some of them that "he was sick and ought to be in bed;" returned about noon, called at my office to see me, I was absent, and he went home, ate no dinner and went to bed sick, appeared in great distress, vomited two or three times in the course of the afternoon, very thirsty, cramp in his limbs, frequent sighing, made an effort to pass his water without success, swelling of the face a little increased since morning. The above particulars I have from himself and his family: at about five, P. M., he sent for me. I went to him immediately, found him in bed, right side of his face opposite the inferior jaw a little swollen and *severe* pain in it; great distress at his stomach, constriction of the throat, difficult swallowing, and labored respiration, pulse 130, lips parched, great thirst, chilly, a sensation of stiffness of the whole body, and also of debility, said, "he was fearful he should have convulsions;" great anxiety of countenance, and some stupor of mind, I inquired what was the matter, he replied, "I have had arsenic in my tooth, and am poisoned." Nine o'clock—had during the evening frequent vomiting, thirst and distress at stomach aggravated, articulation indistinct, and no mitigation of any symptom—one o'clock Tuesday morning, symptoms generally more severe, cold chills frequent, pulse 135, by the active use of such remedial agents as his case required, he became more quiet at about three o'clock, A. M., and soon after fell asleep and slept about an hour, awoke somewhat relieved, retained a little food on his stomach. At eight o'clock, A. M., pulse 130, had felt no disposition to spasms since three o'clock, had passed his water twice during the night with great muscular exertion. Other symptoms as in the evening previous, except the swelling of the face increased, at about ten o'clock his face commenced swelling alike on each side, and swelled with great rapidity, respiration much more laborious than at any other time, frequent sighing and vomiting, countenance expressive of intense anxiety, and with the exception of spasms, the severity of his symptoms became more and more severe through the P. M.; at five o'clock the swelling extended from behind the mastoid process on each side, far up on the temporal and frontal bones, and around his neck, filling it out to the chin and about the face so as to almost close his eyes. Lips greatly distended, parched and livid, vomiting almost incessant, thirst unquenchable, pulse irregular, distress at stomach great, and copious secretions in the throat, at about eight o'clock the swelling of the face appeared to be arrested, but no farther mitigation of his symptoms, he passed the night without any favorable change, or getting any relief, towards morning the powers of his system appeared to be yielding, efforts to vomit were not so effective and were less frequent, swelling of the face a little less, pulse quite irregular, under the influence of stimulants, which he would retain for a short time, he would revive a little but soon sink, he continued much the same except sinking more and more until about eleven o'clock, Wednesday morning, when he died.

I have, I believe, stated all the facts of any importance in relation to the case of Dr. Wolcott, unless it is in relation to the state of his bowels. On Tuesday morning after becoming more quiet he took castor oil, and I dont know that he vomited it up; about four o'clock in the P. M., he had a movement, but the stool was thrown away without being examined, on Wednesday morning he made an attempt to have a movement, but without success, his bowels were considerably distended, when he died. I was with him most of the time from Monday evening, until he died, and the statement above is a true history of his case.

I will further state, that some five or six years since, Dr. Wolcott by using arsenic in preparing the skin of an animal for stuffing became poisoned, and had extensive swelling of his face, neck and hands. In his last illness he frequently told me that "he was now poisoned by arsenic," called for antidotes, &c., and did not appear to have any doubt but that the arsenic which was applied to his tooth, was the sole cause of his illness. After his death, the dentist showed me the tooth, and told me that "the arsenic was placed directly on the exposed nerve," and upon examination, I saw that the tooth was decayed fully down to the hole, through which the nerve passed. He also informed me that the substance applied, was composed of arsenic wet with creosote sufficiently to form a paste.

Believing that the above will be a satisfactory answer to your interrogatories, I remain your obd't servant,

R. M. Woodruff.

Enclosed in Dr. Woodruff's letter, was the following from Mr. Oliver Wolcott, nephew of the deceased, who was requested to make a statement of the facts known to him personally, by Geo.

C. Woodruff, Esq., postmaster of the village, who has kindly contributed to the investigation of this case.

Dr. Brown,

I have been requested, among others, by Mr. George C. Woodruff, to state what I know respecting the symptoms of my late uncle's case. All the information which I possess on the subject I have given to Dr. Woodruff, together with what I obtained from Millie, a faithful old colored woman, who lived with us. I have seen a letter which Dr. Woodruff intends sending to you, containing the symptoms, &c. I do not hesitate to say that it is entirely correct. I was with my uncle during the whole of his illness; he called me up on Saturday night for some laudanum, which he put into his tooth, and soon fell asleep; I sat up with him from Monday, five, P. M., until Tuesday, seven, P. M., when, being perfectly exhausted, I was relieved. His health, during the eighteen months that I have been studying with him, was never better than during the summer and fall of this year. He rose very early, worked in the garden, &c. and congratulated himself on his good health. There was no previous indisposition, to the tooth-ache of Saturday night, that I am aware of, at least I heard no complaint whatever. In conclusion, let me say that the facts are all embodied in Dr. Woodruff's letter, in whom I place the greatest confidence, and who has been my uncle's physician for six years, and who was well acquainted with his constitution, treatment, &c. I remain your obd't servant, Oliver Wolcott.

P. S. Perhaps it would not be amiss for me to say that my uncle told me the same as he did Dr. Woodruff, or nearly so, "that arsenic was the cause of his illness," (which he also told one other person.) This he told me on Tuesday morning.

The following letter, from Mr. Merriman, the respectable dentist of the village of Litchfield, accompanied the two preceding.

*Litchfield, January 3d,* 1844.

Dr. Solyman Brown,

Dear Sir—The following statement of facts, which came under my notice, in relation to the death of Dr. John S. Wolcott, I have already submitted in substance to George C. Woodruff, Esq., by his request; but I will now give a few more particulars in relation to the tooth, and the doctor's conversation with me. Supposing, from your letter, that possibly it might not have reached you, I cheerfully give my testimony to the dental profession, knowing that my course will be approved or not as it shall truly deserve.

On Sunday, November 19th, about one o'clock, P. M., Dr. Wolcott called on me, laboring under a severe tooth-ache which he had had at intervals for the last fortnight, at the same time complaining of flying pains in his limbs and stomach, occasioned (as he said) by a cold which he had taken while superintending his outdoor affairs. At his request, I applied a preparation composed of two parts arsenic and one of morphine, in powder, which had been slightly moistened with creosote a fortnight previous, but nearly dry at this time. He made some inquiry respecting the mixture, and appeared satisfied with my answer. The two right bicuspides of the inferior maxilla were decayed between their adjoining surfaces, and partly broken away, leaving a space of a line at the top of the crown and half a line near the gum, the seat of pain being in the second bicuspid, with the cavity facing the first, above the gum, and sufficient space to allow of inspecting the cavity and its contents with my eye. The two molares next this tooth had been extracted, and the jaw and gums had every appearance of health. It seemed desirable to save the remaining teeth, and I commenced removing the soft portion of bone lying over the nerve, that the arsenic might act quickly upon it; but from its increasing the pain, was obliged to leave it but partially cleansed of soft bone, though it was quite dry from moisture. A piece of cloth being laid around to cover the exposed parts of the gum, I inserted about one-twelfth of a grain of the preparation into the cavity, upon a small pointed instrument. It was then covered with wax perfectly free from air or moisture, or contact with the gum, and as a further security I placed a wedge of wax between the two teeth, cautioning the doctor not to eat upon this side of his jaw. He inquired what time would be requisite to give relief, and I said four or five hours, or before night. He told me that he should give up and place himself on the invalid list, if he had not so much to attend to that he could not afford to be sick, and then left to attend to his professional duties. In the evening, about seven o'clock, he returned to my house, complaining that there was no alleviation of his pain. Both the first and second call were made in presence of my wife and her sister, and this time he seated himself by the table, on which a light was burning, and made some prescriptions for Mrs. M., who had been ill some days with cankered mouth and throat, with slight swelling of the glands. The doctor was in the sitting room near half an hour, the room was warm, and he amused us by his gestures and remarks, which were grave and witty alternately, as a spasm of pain or freedom from it predominated, at the same time making with his knife a swab to use for Mrs. M's throat. In the course of these remarks, were the following, more particularly worthy of notice. Said he, "If you had seen the old gentleman (meaning himself) the other morning, you would not have known him, for when I looked in the glass I did not." "He was a beautiful looking man," making at the same time a gesture with both hands around his face, signifying fullness or swelling. His object in waiting was partly to allow time for the arsenic to act upon the nerve, and, no doubt, in part also from the unwillingness every person manifests to be dismembered of a tooth. Before seating himself in my office, which was destitute of a fire on Sunday, he removed from his neck a large shawl, and laid aside his overcoat and coat, as was his custom in such cases previous. On his holding the light, I examined the mouth, noticed that the wedge of wax was gone from between the teeth, but found the wax over the cavity, and removed it and also the arsenic in a careful manner; it appeared of the same amount and dryness as when applied. There was no sign of abscess or inflammation about the teeth. After cutting the gum, I stood on the left side of the patient, raised my arm over his head, and extracted the tooth with a pair of straight forceps. While the doctor was spitting and rinsing off the blood, I examined the tooth, and found the soft bone between the arsenic and nerve. On lifting it with an excavator, a very slight opening could be made by force into the centre of the tooth, three-eighths of an inch long, and admitting the edge of a knife. No sign of disease like abscess was to be seen on the point of the root. But now, for the first time, the doctor said "he was relieved," "was a new man," &c. and "thought he could sleep to-night." After rinsing his mouth well with cold water, and taking his things, he left, much satisfied with the result; was in my office perhaps half an hour in all. The next Monday afternoon, twenty-six hours at least from the time of application, he called me into his office, and requested the extraction of another tooth, viz. the first bicuspid before mentioned. But I declined this, and asked him if he slept any the night previous, as he expected. He said "no, he had not slept for three nights, and he could not stand this pain." "What shall I do then?" I told him his case needed the doctor more than the dentist, for he owned to exhaustion from want of sleep, riding in the winds, fatigue of mind and body, together with wet feet on Friday, and tooth-ache, combined with pains about his limbs, back and stomach, and that, however reluctant, he would have to rest, and procure sleep and quiet by anodynes and local applications. At length he determined to try these remedies; but suggested a resort to the forceps if they were unsuccessful. I heard no more from him till the evening of Tuesday, when I was informed it was the opinion of his physicians that he could not long survive. He died on Wednesday, A. M. Respectfully yours,

R. B. Merriman.

P. S. When I learned that it was rumored the doctor died from application of arsenic, I requested a post mortem examination, and I am very sorry this request was not acceded to. R. B. M.

Dr. Beckwith has read the above statement, but on account of ill health I am unable to see the other physicians. R. B. M.

The next communication which I received on the subject, is from Dr. J. G. Beckwith, and is as follows:

*Litchfield, Dec. 25th,* 1843, 9 o'clock, P. M.

S. Brown, A. M.

Sir—Your favor of the 22d inst. has just came to hand and I avail myself of a private opportunity by Mr. Perry, a medical student, who goes to the current lectures at the University in the morning, to say to you, that I will investigate the facts in relation to the late death of Dr. John S.

Wolcott, with pleasure; in the meantime, would say that it will appear on investigation, that his death was not in any way attributable to arsenic as an agent, that the quantity used was too small to have produced death ,even if taken into the stomach, that it was carefully applied by the dentist, and covered with wax, that he removed the wax and the filling, a few hours after the application, and extracted the tooth. The doctor being about until the afternoon of the next day, in the meantime exposing himself to the weather, and attending to his profession, and that neither locally nor constitutionally did he exhibit the legitimate effects of arsenic upon the system.

If it should be desirable, a statement can be made out, and would be signed by most if not all of our most respectable physicians, who saw him during his sickness, substantiating the above facts, and exhibiting cases where death has occurred with symptoms very similar to the doctor's, where no arsenic had been used.

The Litchfield paper contained no such statement, and how it first obtained publicity in the Hartford Journal, from which it was copied into almost all the newspapers of the country, we cannot ascertain. The report was of a similar nature, with the paragraph which was found in almost all the papers in relation to the death of Dr. Boardman of Hartford, which I found on investigation, to be probably as little attributable to creosote, as Dr. Wolcott's to arsenic. His death also occurred from extensive and malignant inflammation of the cellular substance about the face and neck, of an erysipelatous character.

I deem it highly important that the facts should be made to appear, and if arsenic is very dangerous in its application, let its use be suspended. You, however, are well aware that the quantity necessary to destroy the sensibility of a nerve of a tooth is entirely powerless, as a poison upon the human system; but I am extending my remarks too far upon this subject at this time, I am very glad that you have been prompted to investigate this subject and believe that the investigation will make it appear that the article in question has been unjustly charged with the death of brother Wolcott. Any assistance I can render you in this investigation will be done cheerfully, please say how far you would like to extend the length of the article, whether a certificate from the physicians would be desirable or not.

In great haste, your friend and servant,

J. G. BECKWITH.

In a subsequent letter, Dr. Beckwith responds to my specific interrogatories, in the following explicit manner:

1st, as to the state of Dr. Wolcott's health? The doctor had previous ill health, as you will learn from the enclosed paper; was subject to erysipelatous attacks, and I once attended him, about two years since, when he was prostrated by an attack of neuralgia, commencing with tooth-ache, and relieved by the extraction of the tooth, after having been confined several days to his bed.

2. As to the length of time previous to the extraction?

You will notice the swelling of which he spoke, at his interview with Dr. Merriman, on the preceding Friday, it must of course have been some days antecedent to this time.

3. As regards the size of the cavity?

Dr. Merriman's letter is very explicit.

4. Was the wax removed by Dr. W., and then restored?

Probably not; as the wax would not have been found by the dentist in the same situation as he placed it, if it had been removed, and the arsenic would not have been found entirely dry if the cavity had been opened. The wedge of wax, spoken of by the dentist, was probably removed, as it was not found when the tooth was extracted; or the filling in the other tooth which pained him might have been removed, if the doctor himself had filled it. We learn from Dr. M's statement, that he wished another tooth extracted ; he also mentioned to some of his patients, on Monday, that he should feel much better after another tooth was removed.

5. Did Dr. W. eat any thing while the wax was out?

This answer is embraced in the last answer to the last question.

6. As to the quantity of arsenic and morphine, and the situation of the cavities?

The communication is full and explicit on this subject; also Dr. Merriman's statement.

7. Were the symptoms manifested during the patient's illness, such as to indicate poisoning by arsenic?

We suppose that this question is fully discussed in the enclosed paper.

8. What medicines were administered by the attending physician?

No medicines, as I could learn, which had reference to the supposition that he was poisoned by arsenic.

9. Can arsenic, acting homœopathically, in so small a dose, destroy life, if all had been taken into the stomach?

The concurrent testimony of all text books on materia medica goes to show the safety of this quantity, as they always recommend the minimum dose, and a quantity which would always be safe to administer to a person having a peculiar susceptibility to the unfavorable operation of a medicine which might have an unfavorable or poisonous effect. This is discussed in the enclosed paper.

10. As to the fact of Dr. W's being formerly poisoned by dressing a skin is not referred to in the enclosed paper?

I was acquainted with the circumstances attending this poisoning, which was merely a superficial affair; by rubbing it on his forehead, he had an eruption, and the fumes had an unpleasant effect upon his olfactory nerves. It occurred a number of years since, and could have no influence in laying the foundation for a more violent effect of the poison upon the system at this time. Mr. E. B. Webster, of this village, assisted the doctor at the time, and corroborates the account I have given of the matter.

11. Was the patient himself a competent judge of the actual cause of his sickness and death ?

He was not, probably, from the fact that he had a strong prejudice against arsenic, and was in so much pain and distress, that he could not judge coolly, and perhaps did not know the quantity used ; he did not have any such impression when he last saw the dentist, nor did he impute any blame to him ; but from the violence and fatal tendency of his symptoms, it was not unnatural, nor in any degree surprising, that so potent an article as arsenic, where its tendency is evil, should be regarded as the unseen agent of his sufferings.

12. What other theory can be sustained, &c. as the cause of death?

Discussed in the enclosed paper.

13. As to the quantity which allopathic physicians regard as sufficient to produce death, &c.?

Two grains Dr. Beck mentions as the quantity which has produced it. You will notice in the paper, and other writers make mention of a larger quantity. (Dr. Beck mentions, vol. 2, p. 140, ed. 1823,) Dr. Petit, of Lyons, relates a person who survived after having taken half an ounce of arsenic, and he attributes it to the violent vomiting that ensued; so that if it were possible for the vomiting in Dr. W's case to have been the effect of the poison, it is itself important, as Dr. Beck mentions, in the same volume, (p. 140,) "I apprehend that the patient's vomiting or not vomiting has the greatest influence on the cause and variety of the symptoms: in the former case, the poison may be rejected before it has time to produce injurious effects, while, in the latter case, death may be inevitable; and again, the poison taken, may, from its quantity, or some other reason, itself produce vomiting, and thus prevent the fatal termination." He then adduces the case related by Dr. Petit.

14. What evidence that some of the poison was left in the tooth until it was extracted?

Dr. Merriman mentions in his statement, that he found it all there and dry, the best evidence in the world.

The certificates of Drs. J. G. Beckwith, Benjamin Welch, Jr. and Wm. Buel, all of the native village of Dr. Wolcott, and medical gentlemen of undoubted judgment and veracity, conclude the evidences in this interesting case.

*Litchfield, Jan'y,* 1844.

TO SOLYMAN BROWN, M. D.

Dear Sir—The disease which proved fatal to the late Dr. John S. Wolcott of this village, was so distressing and malignant in its character, and so sudden was the lamented result, that it might well lead to inquiry as to its nature and origin; and when it was known that an agent so poisonous as arsenic had been applied with a view to destroy the nerve of a painful tooth, and that he himself entertained apprehensions that this was the cause, we might expect that a report, that such was the fact, would readily gain credence. That Dr. Wolcott entertained such apprehensions, is neither surprising or unaccountable, when we consider the influence of imagination excited by disease and suffering. It cannot be made to appear, how entirely without foundation in truth, is the opinion that arsenic was the cause of death, without a full and unreserved statement of facts in the case.

For a history of the symptoms to the time we visited the patient, we are principally indebted to Dr. Woodruff, the attending physician. He has communicated to you a statement of the facts as witnessed by himself. Dr. Welch first visited the patient on Tuesday evening, Nov. 21st, and remained in attendance, at the request of Dr. Woodruff, through the night. Dr. Beckwith saw him early the following morning.

When visited Tuesday evening, the whole face and neck were excessively swollen, red and painful, and the swelling extended downwards upon the upper part of the chest. Upon the sides of the neck, and in the region of the lower jaw, especially upon the right side from which the tooth was extracted, the swelling was hard and painful under pressure. The tumefaction of the face was so great, as nearly to close the eyes. The mouth could not, without difficulty, be sufficiently opened to protrude the tongue, or to allow an examination of the tongue and fauces. The patient spoke with difficulty, and then scarcely intelligibly. Deglutition was difficult and painful. Respiration laborious, and attended with mucous rattle in the throat, and the patient was frequently, by drinking warm water, irritating the fauces with an instrument, and by voluntary efforts, exciting vomiting, apparently with a view to dislodge the mucus, as he seldom or never vomited until some or all of these means were employed; the skin was cool and moist; pulse too feeble, irregular and frequent to be counted; great restlessness, and frequent tossing of himself about; made no complaint of pain in the stomach or bowels, or of tenderness under pressure, upon the abdomen; he has had several motions of the bowels by stool, the effect of a cathartic, muscular strength was sufficient to raise and support himself, upright in bed, and to support himself when helped from the bed with little assistance; voids his urine without any expression of pain or difficulty; no apparent delirium.

The treatment adopted, at this stage of the disease, and under circumstances so apparently desperate, can afford but little light in regard to its tendency, and therefore need not be minutely described. It was evident that the effusion of matter, if suppuration occurred, would not be preceded by adhesive action to prevent the matter from spreading, and would be attended with extensive sloughing of the cellular membrane. It appeared more probable that the patient must speedily sink from irritative fever, and exhaustion. Our only hope was in immediately arresting the tendency to suppuration, at the same time that we afforded the necessary support and quiet to the system. Several leeches were accordingly applied to the neck, and the discharge of blood, promoted by cloths wet in warm water; also at the solicitation of the patient, the scarifier and a cup, two or three times repeated, were applied in the vicinity of the inflamed part. After this, the whole fore part and sides of the neck, and lower part of the face were brushed over with tincture of iodine. Internally small doses of tartarised antimony, and sulphate of morphine, and also Dover's powders, with one or two grains of sub-murias hydrarg., were administered; brandy and water, and other diffusible stimulants were allowed; sinapisms, and jugs filled with hot water, and warm flannels, were applied to the extremities. The patient was repeatedly cautioned as to the injurious tendency of so frequent, voluntary efforts and irritation to excite vomiting.

After this time, there was certainly no increase or extension of the swelling or inflammation, but rather diminished redness, tumefaction and hardness; the intervals of vomiting were more protracted and extended, from half an hour, to one or two hours. During these intervals, his medicine and such articles of drink and nourishment as were given him, were retained upon the stomach, and in moderate quantity; did not appear to excite uneasiness, but the patient gradually became weaker, efforts to swallow more painful, and in the morning, were attended with apparent sense of strangling. Thus retaining his reason till near the last, he expired at about 11 o'clock, on Wednesday, A. M.

To the intelligent dental practitioner, or the medical and surgical professions, the above statement, we believe, is all that is necessary, when the time, quantity and manner, in which the arsenic was applied, is known, but as you request us not only to communicate the facts, as witnessed by ourselves, but a history of the whole circumstances of the case with our opinion in regard to it, we will add the following statements, which are at your service, to dispose of as you think proper.

The disease was evidently erysipelatous inflammation of the neck and face, with tendency to diffuse suppuration in the cellular substance. We think there is fortunately sufficient evidence as to the true origin of the disease, independently of any influence arising from the action of arsenic.

1st. There is abundant evidence of predisposition arising from the previous state of the patient's health. He had, more than once, attacks of erysipelas; the Friday previous to his last illness, in conversation with his friends, he jocosely remarked, that "had they seen this gentleman in the morning, they would not have known him, as he did not himself when he looked in the mirror, his face being so swollen." On the next day, in an interview with another, he repeatedly exhibited evidence of double vision. His house keeper says, that for two or three weeks previous, he had been afflicted with inflammation upon his limbs, requiring poultices, &c. *which had, at the time of his illness, healed.*

2dly. The extraction of a tooth, in the forming state of inflammation of the jaw, is known, many times, to be followed by very serious aggravation of the disease, and even fatal consequences have resulted.

3dly. The day succeeding the extraction of the tooth, and when there can be little doubt that inflammation and fever, already existed, he rode several miles, in windy and uncomfortable weather, to visit patients. Exposure to cold is alone sufficient to excite fatal erysipelatous inflammation in the predisposed, and such inflammation is especially dangerous when seated in the neck and throat.

These circumstances it is contended, are fully sufficient to account for the disease. In illustration of our sentiments we may be allowed a single quotation. "The erysipelatous more than any other inflammation, following injuries, connects itself with peculiarities of constitutions, and gives the impression, from the seeming inadequacy of the exciting cause, and the sudden, rapid and destructive character of the disease, of its originating from some specific irritation."*

We will state as concisely as possible, the direct evidence that the disease was not owing to the poisonous action of arsenic applied to the tooth.

We are not aware that any one who has with any intelligence examined the subject, supposes that the poison escaped from the tooth, and was inadvertently taken into the stomach. There is not the slightest evidence, either from the circumstances of the case, or the subsequent symptoms, to indicate that such was the fact. The tooth affected, and into which the arsenical preparation was introduced, was a bicuspis of the lower jaw, with a favorable cavity for the introduction of the remedy, and effectually securing it there, by a covering of wax, and we have no reason to doubt, the whole was judiciously, and skilfully performed by the dentist. We have no proof that any escaped so as even to fall upon the tongue, or mucous membrane of the mouth.

The attack of pain in the stomach and vomiting, referred to by Dr. Woodruff, did not occur until more than thirty hours after the application,

* Erysipelatous disease is frequently epidemic. An instructive example occurred at Plymouth, Eng., and extended to the adjacent country, in the summer of 1824. Of fifteen cases which occurred at Plymouth dock-yard, and are related by Dr. Butler,* twelve were fatal. In all these cases, the disease was excited by local injury, which served as a starting point to the inflammation. An epidemic erysipelatous disease has prevailed extensively, in the north and west parts of the state of Vermont, the last two or three years, and has been described by J. A. Allen, M. D.,† of Middleburg, and published in the periodicals of the time, and by C. Hall, M. D., of Burlington, Vt. and G. J. Dexter, M. D., of Lancaster, N. H. in the last January number of the American Journal of Medical Sciences. In this connection it may not be altogether unworthy of notice, that Dr. Wolcott, about four weeks previous to his death, attended a case of diffuse inflammation seated upon the thigh, and about the hip, which proved suddenly fatal. Also at the time of his death, two persons upon whom he had attended, were laboring under epidemic sore throat.

* See Review of Butler's Remarks on Irritative Fever, &c. in American Medical Review and Journal, vol. 2, p. 325.

† See Topaz, published at Middleburg, March, 1842.

and twenty hours after the tooth was extracted, and the patient had, in the interval, repeatedly taken food. Could the operation of so virulent a poison be thus delayed, how tremendous would be the agent, for evil, in the hands of the designing. In cases of poisoning, we should have to inquire into the circumstances, not of the last, but of several previous repasts; there was an absence of symptoms of inflammation of the stomach and bowels, which must have existed, had a poisonous dose of the article been received into it, nor could the inflammation of the neck and face be at all explained upon such a supposition.

The whole quantity applied to the tooth, was not sufficient, if received into the stomach, to cause death. It did not exceed the ordinary dose as exhibited in intermittents, lepra, &c., that is, from one-tenth, to one-fourth of a grain; the dose has been increased when administered internally in the form of Fowler's solution, even to one-half of a grain with beneficial results.* The dentist tells us that from one-eighth to one-twelfth of a grain of the arsenious acid, with half the quantity of morphine, moistened with creosote, is quite sufficient to destroy the nerve of a tooth, and is more than was used in the present case, the tooth having but a single root.

The more plausible supposition is, that the consequences resulted from the local action of the remedy. Cases have occurred showing peculiar susceptibility to its influence. The external, as well as the internal application of arsenic is, in sufficient quantity, known to be poisonous. But here, if strictly confined to the cavity of the tooth, it is believed there could be absolutely no absorption; if it escaped the tooth at all, there must have been the protection afforded by the lining membrane of the mouth, and skin covering the part through which it could only act slowly, and after the membrane was destroyed. Such a supposition assumes an augmentation of the susceptibility of the patient, extraordinary to a marvellous extent, and altogether beyond the reach of our credulity. We are not so fearfully made that an irritating agent like arsenic, is more fatal when applied to the surface of the body, undenuded of its covering, than to the peculiarly sensible and vital organ, the stomach.

Not only all theory, but all experience, is opposed to such a view of the case. In the multitude of instances in which it has been applied, in the same manner, by dentists, no such consequences have heretofore followed. The late Prof. Hosack, of New York, recommended it in his public lectures, as the most appropriate remedy for the destruction of common warts, in cases where the cicatrix resulting from its action would not be objectionable. For this purpose, many times the quantity necessary for the destruction of the nerve of a tooth would be required. Combined with opium and other substances, it is often applied, by the surgeon, directly to ulcerated surfaces, to change action in cases of lupus or cancerous ulceration, with entire safety

* Prof. Beck has the following note. See Medical Jurisprudence, vol. 2, p. 181. "The smallest quantity of arsenic may prove injurious, and it is said that *two* grains have caused death."

and success. It is generally esteemed a safe and effectual agent for the removal of cancerous tumors of small size, where there are objections to the employment of the knife; and it possesses the peculiar advantage of not extending its operation upon the surface beyond the part to which it is applied. It is also the ingredient which constitutes the principal active agent in most of the cancer plasters and nostrums, so indiscriminately employed by empirics, and danger arises, in these cases, only from the continued or too often repeated application of the poison, in such quantity that it acts as a caustic.

There are also circumstances, in the case of Dr. J. S. Wolcott, which we believe distinguished it from poisoning by the external application of arsenic, quite independently of the quantity and the manner in which it was applied.

We have already trespassed so largely upon your patience, that we will direct attention only to a single point; this is, the disproportion in the amount of local inflammation, and disturbance of the nervous and digestive systems, which exists in the two cases. In the former, the malignity of the disease is marked, especially, by the intensity and extent of the erysipelatous inflammation, attended only by the ordinary symptoms of irritative fever, and, so far as we had an opportunity to judge, an amount of gastric disturbance not unusual in the disease. In the latter, there is either an entire absence of external inflammation, or it is in no measure proportionate to the violence or malignity of the general symptoms; which are characterized by paleness, faintness, paralysis, tremors, convulsions, disturbance of the intellectual faculties, coma, &c.

We are told that the symptoms of poisoning by arsenic "are similar, in all essential circumstances,"* whether it is taken into the stomach or applied to a wound. Dr. Jaeger, of Stuttgard, in a summary of results derived from many experiments on animals, observes, that "when applied to the sound skin it seldom injured it. If applied to a wound, it never, after death, was observed to be gangrenous or *inflamed*, was *rarely swollen*, but *generally pale*."† The preparation employed in his experiments was chiefly a solution of the arsenious acid in water, in the proportion of one to sixteen.

Respectfully, yours,

Benj'n Welch, Jr.
J. G. Beckwith.

Having visited Dr. Wolcott in his last sickness, in company with Drs. Welch and Beckwith, and having endeavored, at the time, and since, to obtain information from other sources, of the various circumstances of the case, and now having attentively perused the above statement, I discover no reason for apprehending that his sickness and death were the effects of arsenical poison. Wm. Buel.

* See Beck's Medical Jurisprudence, vol. 2, p. 199.

† Review in Edinburgh Med. and Surg. Journal, vol. 7, p. 80—84.

